# Supplementary material for: Programme death ligand 1 expressions as a surrogate for determining immunotherapy in cervical carcinoma patients
Source: PLoS One. 2022 Feb 9;17(2):e0263615. doi: 10.1371/journal.pone.0263615 (PMC8827435; doi:10.1371/journal.pone.0263615)
Supplement: Attachment — Submitted filename: Response to reviewers..docx [file pone.0263615.s001.docx]

Dr. Sebastian Omenai

Dept of Anatomical Pathology

Edo State University.

The Editor,

PLoS One

Sir/Ma

**Re: Programme Death Ligand 1 Expressions as a Surrogate for Determining Immunotherapy in Cervical carcinoma Patients**

We sincerely appreciate your time and effort in improving our manuscript with the criticism and corrections. The following are our responses to the comments and suggestions of the reviewers.

Reviewer #1.

We have made some spelling and grammar corrections in the manuscript just like suggested. The number of cases stained and scored as well as the PD-L1 clone used are now mentioned in the abstract. We have added more literature on the use of PD-L1 in cervical carcinomas especially in regards to prognostic implications of PD-L1 expression. Truly, PD-L1 is just one of the immune evasion mechanisms of cancers currently being studied. Our write up initially inadvertently presented PD-L1 as the only mechanism, which we have corrected in the manuscript. We agree that using non diagnostic PD-L1 clone is a major drawback, we had difficulty sourcing for the more popular clones for our laboratory. We have expressed the limitations this pose to our findings in the manuscript. We reviewed the slides and used the combine positive scoring system which takes note of positive infiltrating myeloid cells in determining PD-L1 status.

Reviewer #2.

We have recalculated the PD-L1 status using the CPS. We didn’t use 22C3 or SP142 clone assay in our study. Hopefully we can get dealers to supply to our laboratory as soon as we establish the need for PD-L1 immunohistochemistry in our society. We have stated the non-diagnostic genetex PD-l1 assay we used and the subsequent limitations it poses.

Thanks,

Yours sincerely,

Dr. Sebastian Omenai
